# Diffusion Models for Neuroimaging Data Augmentation: Assessing Realism and Clinical Relevance

**DOI:** 10.1007/s10916-025-02300-1

**Published:** 2025-11-17

**Authors:** Giulio Mallardi, Fabio Calefato, Filippo Lanubile, Giancarlo Logroscino, Benedetta Tafuri

**Affiliations:** 1 Department of Computer Science, University of Bari, Bari, Italy; 2 Department of Translational Biomedicine and Neuroscience, University of Bari, Bari, Italy; 3 Center for Neurodegenerative Diseases and the Aging Brain, University of Bari at Pia Fondazione “Card. G. Panico”, Tricase, Italy; 4 Department of Engineering of Innovation, University of Salento, Lecce, Italy

**Keywords:** Diffusion models, Synthetic MRI generation, Rare neurological diseases, 3D Medical image synthesis

## Abstract

Data scarcity remains a major obstacle to the application of deep learning techniques in medical imaging, particularly for rare neurodegenerative diseases. This study investigates the use of denoising diffusion probabilistic models (DDPMs) to generate synthetic 3D T1-weighted brain MRI images in this context. Addressing the dual challenges of limited training data and structural fidelity, we propose a generative pipeline trained on a multicenter dataset of healthy subjects. The model suggests the potential to produce anatomically coherent synthetic scans with realistic variability. Quantitative evaluation based on Maximum Mean Discrepancy confirms the similarity between real and generated data distributions, while visual assessments highlight the preservation of global and local brain structures. Despite limitations in high-frequency detail reconstruction, the results suggest that DDPMs hold promise as a tool for augmenting neuroimaging datasets and supporting downstream tasks such as classification and segmentation. This work lays the foundation for future research aimed at improving resolution and adapting generative models to the specific challenges of rare disease imaging.

## Introduction

Magnetic Resonance Imaging (MRI) is a cornerstone technique for non-invasive investigation of brain structure and function. It has significantly advanced the understanding of neurological disorders by enabling the visualization of detailed anatomical features. In the context of rare neurodegenerative diseases such as Amyotrophic Lateral Sclerosis (ALS) and Frontotemporal Dementia (FTD), however, research progress is hindered by a persistent lack of large-scale, high-quality neuroimaging datasets. This limitation stems from the inherently low prevalence of these conditions and the associated challenges with data collection, harmonization, and privacy regulation. As a result, both research and clinical efforts suffer from limited statistical power, poor model generalizability, and potential diagnostic uncertainty.

To address this bottleneck, recent developments in machine learning have introduced generative models as a viable strategy for data augmentation. Among these, diffusion models have emerged as a particularly promising solution due to their ability to learn complex, high-dimensional data distributions and generate high-quality synthetic samples. When integrated into the neuroimaging analysis pipeline, such models offer the opportunity to expand training datasets in a meaningful way, potentially enhancing both the robustness of machine learning models and the interpretability of clinical findings.

In this study, we explore the use of denoising diffusion probabilistic models (DDPMs) to generate synthetic three-dimensional MRI from healthy subjects, with the broader aim of supporting data-driven approaches in rare neurodegenerative disease research. We hypothesize that diffusion-generated images can approximate the statistical and anatomical characteristics of real MRI scans, thus serving as a high-fidelity resource for training and validating automated diagnostic systems. If successful, this approach could not only improve the reliability of neuroimaging studies but also contribute to the clinical management of disorders like ALS and FTD by enabling the development of more robust decision-support tools.

The remainder of this paper is organized as follows. Section “Background and Related Work” reviews prior work on generative models for medical image synthesis, with a focus on diffusion models. Section “Proposed Approach” presents the details of our synthetic image generation pipeline. Section “Experimental Setting” outlines the experimental setup, including datasets and preprocessing procedures. Section “Results” reports and analyzes the results. Section “Discussion” analyzes the results, discusses limitations, and outlines key challenges and opportunities in applying diffusion models to rare disease imaging. Finally, Section “Conclusion and Future Work” concludes the paper and outlines future directions for clinical integration and model refinement.

## Background and Related Work

In this section, we provide an overview of the key generative modelling paradigms applied to medical imaging, with an emphasis on brain MRI synthesis. We cover both foundational models and recent innovations, setting the theoretical background and empirical context for the approach proposed in this study.

Generative modelling has progressively established itself as a cornerstone in medical image synthesis, enabling a wide range of applications, including data augmentation, privacy-preserving image generation, and simulation of pathological scenarios. Among early generative frameworks, *Variational Autoencoders (VAEs)* and *Generative Adversarial Networks (GANs)* have played a pivotal role in shaping methodological developments. Recent architectural refinements, such as *StyleGAN2-ADA*, have markedly enhanced the visual realism of synthetic T1-weighted brain MRI slices, to the extent that expert raters report minimal perceptual differences between real and generated images [1].

While GANs have demonstrated remarkable success in synthesizing visually convincing samples, they are often challenged by instability during training, sensitivity to hyperparameter tuning, and the well-known issue of mode collapse. Furthermore, the adversarial loss function used in GANs lacks a probabilistic interpretation, which complicates their use in downstream tasks requiring likelihood estimation or uncertainty quantification. Diffusion-based approaches address many of these limitations by offering a more principled probabilistic formulation. *Denoising Diffusion Probabilistic Models (DDPMs)*, as introduced by Ho et al. [2], rely on a stochastic forward-noising process and a learned reverse-denoising mechanism, achieving state-of-the-art results in natural image generation. These principles have been further optimized in *Latent Diffusion Models (LDMs)*, proposed by Rombach et al. [3], which operate within compressed latent spaces of autoencoders to reduce computational overhead while preserving high-fidelity outputs.

The medical imaging community has rapidly adapted these paradigms to clinical data. Khader et al. [4] demonstrate that combining DDPMs with VQ-GANs yields anatomically coherent 3D medical volumes, outperforming standard GANs in both fidelity and variability. Similarly, Dorjsembe et al. [5] introduce Med-DDPM, a conditional framework that synthesizes brain MRIs from segmentation masks, providing compelling use cases in data anonymization and augmentation. Further advancing this trajectory, Dhinagar et al. [6] employ diffusion models to generate counterfactual MRI scenarios in Alzheimer’s disease, enhancing classifier interpretability and facilitating exploratory neurobiological hypotheses. Complementarily, Pinaya et al. [7] show how conditioning LDMs on demographic and anatomical variables enables the creation of large-scale, privacy-compliant neuroimaging datasets.

Beyond these empirical contributions, novel lines of inquiry are emerging. Peng et al. [8] introduce *BrainSynth*, which uses a metadata-aware DDPM capable of producing structurally plausible 3D brain MRIs across a wide demographic span. Other works explore the synthesis of resting-state fMRI connectivity maps using diffusion-based models, expanding the utility of generative models beyond structural imaging [9]. The concept of pseudo-healthy image generation has also gained traction, with recent GAN-based approaches applying contextual inpainting to pathological regions to simulate healthy anatomical structures [10]. Lastly, an emerging body of theoretical work positions generative modelling as a foundational technology for the construction of synthetic patient cohorts and individualized digital twins, with direct implications for personalized medicine and regulatory science [11].

Despite this growing body of literature, several limitations persist. In particular, these observations highlight the persistent lack of generative models that explicitly incorporate anatomical structure, demographic priors, or domain-informed constraints—elements that are crucial to ensure the realism, consistency, and clinical relevance of synthetic neuroimaging data.

## Proposed Approach

**Fig. 1:**
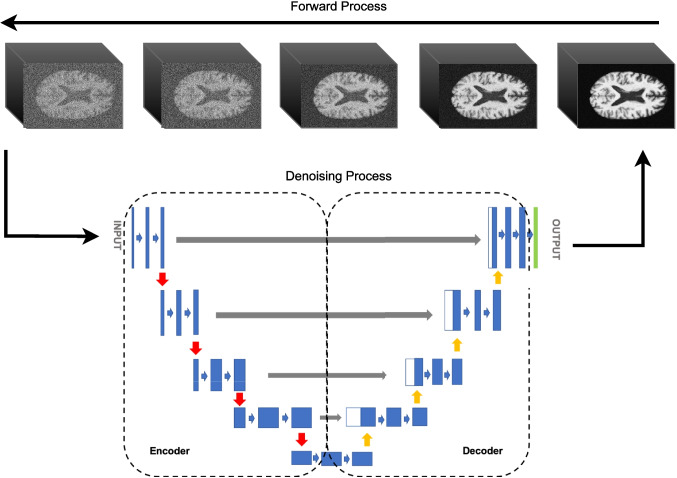
Architecture of the denoising diffusion probabilistic model (DDPM) adapted for 3D brain MRI synthesis

We propose a generative framework based on Denoising Diffusion Probabilistic Models (DDPMs) for the synthesis of high-resolution 3D brain MRI volumes. As illustrated in Fig. 1, the model is trained on preprocessed T1-weighted MR images and learns to generate anatomically consistent synthetic counterparts. The architecture and methodology are tailored to address the challenges of data scarcity and structural variability inherent to rare neurodegenerative conditions. The following subsections outline the theoretical underpinnings, model design, and training configuration.

### Diffusion for MRI Synthesis in Neuroimaging

Diffusion models have emerged as a compelling alternative to GANs and VAEs for generative modeling, offering superior stability and visual quality [12]. In neuroimaging, their application has proven effective for generating synthetic MRI data in domains where clinical datasets are limited and diversity is critical. Prior work by Dhinagar et al. [6] and Pinaya et al. [7] demonstrates their utility for Alzheimer’s disease research and for producing large-scale, high-resolution synthetic brain MRI repositories.

In contrast to GANs, DDPMs minimize mode collapse risk and yield more structurally consistent outputs. Compared to VAEs, DDPMs provide improved image fidelity and better preserve anatomical detail, though they require longer training times and careful noise schedule calibration.

### Opportunities and Challenges in Rare Disease Modeling

The application of DDPMs to brain MRI synthesis presents several promising opportunities in the context of rare neurodegenerative conditions, where the availability of annotated imaging data is often limited. These generative models can augment existing datasets with realistic, synthetic samples, potentially enhancing the training of diagnostic or segmentation algorithms.

A key advantage of DDPMs is their ability to provide interpretable intermediate states, allowing for detailed inspection of the denoising trajectory. This feature can be particularly valuable in rare disease research, where understanding the generative process may yield insights into subtle anatomical alterations. Furthermore, DDPMs facilitate the creation of privacy-preserving datasets, which are critical when sharing sensitive neuroimaging data across institutions.

Nonetheless, the high dimensionality of 3D MRI volumes (e.g., $84 \times 128 \times 84$) combined with the inherent heterogeneity of rare disease cohorts introduces notable challenges. Training stable and expressive models requires both careful architectural choices and robust regularization strategies to prevent overfitting. Moreover, preserving disease-specific anatomical features while ensuring structural plausibility remains a non-trivial task. Sophisticated loss functions and the incorporation of domain knowledge, such as expert-derived anatomical priors, may be necessary to guide the generative process.

### Theoretical Foundations

A DDPM defines a forward diffusion process $q(x_{1:T}|x_0)$ that progressively corrupts the original data $x_0$ into Gaussian noise across *T* timesteps. At each step, noise is added according to:

1 $$\begin{aligned} q(x_t|x_{t-1}) = \mathcal {N}(x_t; \sqrt{1 - \beta _t}x_{t-1}, \beta _t \textbf{I}), \end{aligned}$$

where $\beta _t$ is the variance schedule. This process eventually produces a latent variable $x_T \sim \mathcal {N}(0, \textbf{I})$, effectively destroying the structural content of the input image.

The model learns a reverse denoising process $p_\theta (x_{t-1}|x_t)$ using a neural network $\epsilon _\theta (x_t, t)$ to approximate the noise added at each step. The training objective is to minimize the expected mean squared error:

2 $$\begin{aligned} \mathcal {L} = \mathbb {E}_{t,x_0,\epsilon }[\Vert \epsilon - \epsilon _\theta (x_t, t)\Vert ^2], \end{aligned}$$

where $x_t = \sqrt{\bar{\alpha }_t}x_0 + \sqrt{1 - \bar{\alpha }_t} \epsilon$ and $\bar{\alpha }_t = \prod _{s=1}^t (1 - \beta _s)$. This objective yields a simplified and stable training routine.

At inference time, the network starts from $x_T \sim \mathcal {N}(0, \textbf{I})$ and iteratively applies:

3 $$\begin{aligned} x_{t-1} = \frac{1}{\sqrt{1 - \beta _t}} \left( x_t - \frac{\beta _t}{\sqrt{1 - \bar{\alpha }_t}} \epsilon _\theta (x_t, t) \right) + \sigma _t z, \end{aligned}$$

where $z \sim \mathcal {N}(0, \textbf{I})$ and $\sigma _t$ is a small added noise to ensure sample diversity.

### Model Configuration and Training

The proposed diffusion model was implemented using a customized 3D U-Net architecture within the MONAI framework^1^ [13], which is tailored for deep learning in medical imaging. The network processes input and output tensors of size $84 \times 128 \times 84$, corresponding to downsampled T1-weighted MRI volumes.

The architecture comprises three spatial resolution levels, with channel dimensions set to [128, 128, 256] across the encoding path. Each level includes two residual convolutional blocks. Attention mechanisms, with 256 head channels, are applied at the bottleneck to capture long-range spatial dependencies critical for anatomical coherence.

The noise scheduling strategy was defined using the DDPMScheduler class from MONAI^2^, configured as follows:
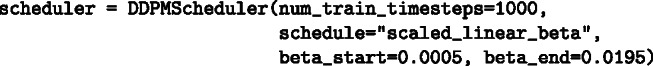


This scheduler generates a linearly increasing $\beta _t$ sequence, scaled for numerical stability, especially over long diffusion chains. For each step *t*, the corresponding $\alpha _t = 1 - \beta _t$ and $\bar{\alpha }_t = \prod _{s=1}^{t} \alpha _s$ are computed, enabling precise control of the variance injected during noise addition and removal.

The model was trained over 400 epochs using the Adam optimizer with a learning rate of $5 \times 10^{-5}$. To optimize training on large 3D volumes, we employed the ‘Accelerate‘ library for efficient multi-GPU training.

Training was conducted on the *LEONARDO EuroHPC Tier-0* infrastructure at *CINECA*^3^, leveraging its Booster Module equipped with BullSequana X2135 “Da Vinci” nodes. Each node includes 32-core Intel Xeon Platinum 8358 CPUs, 512 GB RAM, and 4 NVIDIA Ampere A100 GPUs (64 GB each) interconnected via NVLink 3.0. This configuration allowed us to train diffusion models efficiently on high-resolution volumetric MRI data.

## Experimental Setting

To evaluate the effectiveness and realism of our generative framework, we conducted experiments using a curated collection of high-quality, publicly available structural brain MRI datasets. This section outlines the rationale behind the dataset selection, the preprocessing pipeline adopted, and the configuration used for model training and validation.

### Dataset Details

We initially focused on modeling the distribution of healthy brain anatomy. This approach was chosen to establish a robust generative baseline that captures normal structural variability before extending the methodology to pathological cohorts. Modeling healthy brains represents a necessary foundation for subsequent applications involving rare neurodegenerative conditions, such as Frontotemporal Dementia (FTD), where high-quality annotated datasets are often scarce.

Through our collaboration with the Center for Neurodegenerative Diseases and the Aging Brain, University of Bari Aldo Moro at Pia Foundation of Cult and Religion "Card. G. Panico" (CMND), we assembled a diverse dataset of T1-weighted brain MRI scans drawn from multiple publicly accessible repositories. An overview of the included datasets is provided in Table 1.

**Table 1:** Summary of public brain MRI datasets used in this study

Dataset	Type	Subjects	Target Population	Ref.
ADNI (Alzheimer’s Disease Neuroimaging Initiative)	Longitudinal	819	Mild cognitive impairment, Alzheimer’s disease	[14]
NIFD (Neuroimaging Frontotemporal Dementia)	Longitudinal	200	Frontotemporal dementia, healthy controls	[15]
PPMI (Parkinson’s Progression Markers Initiative)	Longitudinal	600	Parkinson’s disease, prodromal subjects, controls	[16]
OASIS-1	Cross-sectional	416	Healthy aging and dementia	[17]
OASIS-2	Longitudinal	150	Healthy elderly, early AD	[18]
OASIS-3	Longitudinal	1,098	Aging, MCI, dementia	[19]

A total of 1,017 images were selected for training and evaluation. These were randomly split into a training set (80%) and a validation set (20%), ensuring subject independence across the two partitions.

### Preprocessing and Input Representation

To ensure consistency in orientation, contrast, and anatomical alignment across the different datasets, all brain MRI scans were subjected to a standardized preprocessing pipeline based on AssemblyNet [20]. The procedure, adapted from volBrain protocols [21], included the following steps:

1. **Denoising** Manjón et al. [22]: Adaptive non-local means filtering was applied to reduce random fluctuations in voxel intensities, enhancing the signal-to-noise ratio while preserving structural detail.
2. **Inhomogeneity correction** Tustison et al. [23]: N4 bias field correction was used to remove low-frequency intensity non-uniformities induced by magnetic field inhomogeneities, improving intensity homogeneity across brain volumes.
3. **Affine registration to MNI space** Avants et al. [24]: Each scan was aligned to the Montreal Neurological Institute (MNI) standard template using ANTs, mapping images into a common coordinate system (181×217×181 voxels, 1×1×1 mm^3^ resolution), thus enabling inter-subject anatomical comparability.
4. **Fine inhomogeneity correction using SPM** Ashburner and Friston [25]: A second correction pass was performed using the unified segmentation framework in SPM to further refine tissue intensity profiles.
5. **Tissue-based intensity normalization** Manjón et al. [26]: Intensities were normalized based on tissue classes (e.g., gray matter, white matter), enabling consistency across subjects and scanner types.
6. **Brain extraction** Manjón et al. [27]: Non-brain structures such as skull and scalp were removed using a non-local patch-based segmentation approach, isolating the intracranial volume for downstream analysis.

Following preprocessing, all images were refined by normalizing voxel intensities within the brain mask and zeroing the background. This step ensured that brain regions were scaled consistently while eliminating irrelevant background signals.

To accommodate GPU memory constraints associated with training diffusion models, each volume was resized from its original resolution (181×217×181) to 84×128×84. This resizing preserved essential structural characteristics while reducing computational load, a critical factor in enabling efficient training on available hardware resources.

## Results

In this section, we present the results of our diffusion model.

### Model Optimization

**Fig. 2:**
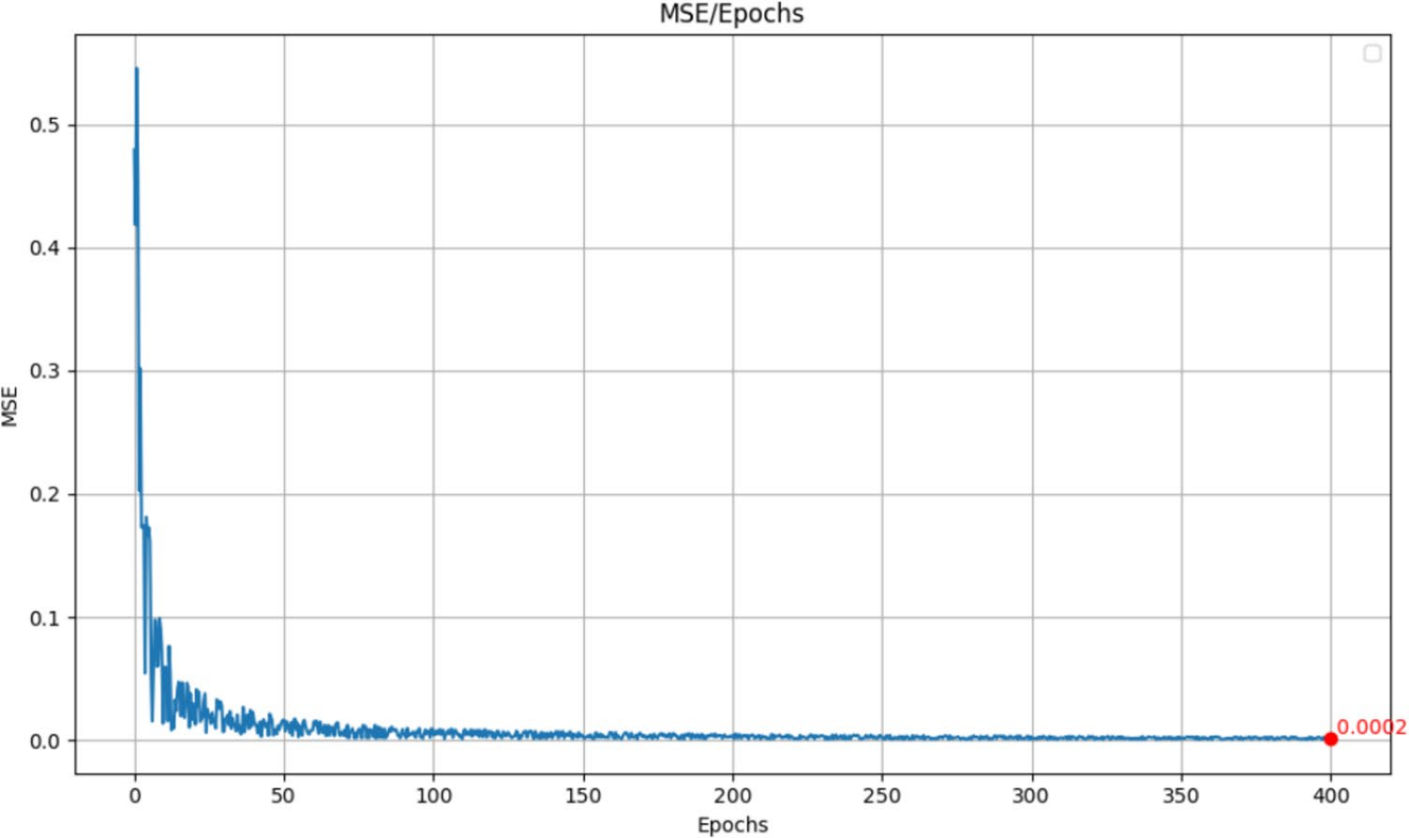
MSE behavior during the training phase in 400 epochs

In the context of diffusion models, Mean Squared Error (MSE) serves as a crucial loss function during the training process, as it quantifies the difference between the predicted noise and the actual noise added to the data at each timestep of the diffusion process. More specifically:

4 $$\begin{aligned} MSE = \frac{1}{n} \sum _{i=1}^{n} (y_i - \hat{y}_i)^2 \end{aligned}$$

where $y_i$ represents the actual noise added to the image, and $\hat{y}_i$ is the noise predicted by the model. A lower MSE indicates that the model has become more adept at predicting the noise added during the forward diffusion process, which is essential for effective image generation during the reverse diffusion process. The training of our diffusion model took about six hours and covered 400 epochs. At this point, we observed convergence in our primary loss metric (MSE). The final MSE value achieved was 0,0002, as illustrated in Fig. 2.

### Evaluation Metrics

Frechet Inception Distance (FID) [28] calculates the distance between two distributions of feature vectors. This metric was explicitly applied to assess the quality of synthetic images compared to real ones. In order to compute the distance, it is necessary to load a pre-trained model (for example, RadImageNet for 2D and MedicalNet for 3D images), which will extract feature vectors from the images and then compute the statistics like mean and variance used to compute the Frechet distance. A lower value of FID means that the two distributions are similar.

Unbiased Maximum Mean Discrepancy (MMD) [29] is a kernel-based method to measure the similarity between two distributions. It is a non-negative metric where a smaller value indicates a closer match between the two distributions. Multi-Scale Structural Similarity Index Measure (MS-SSIM) [30] is a similarity metric usually used in image generation contexts to measure the structural similarity of data within the same dataset. This index is a value between -1 and 1, where 1 indicates perfect similarity, 0 indicates no similarity, and -1 indicates perfect anti-correlation.

We evaluated these metrics on 86 images from both the real and synthetic datasets. To enhance methodological transparency, we report 95% confidence intervals (CIs) for MMD, FID, and MS-SSIM, estimated via 1000 bootstrap resamples of the real and generated sets using a non-parametric approach. Results are shown in Table 2.

**Table 2:** Comparison of MMD, FID, and MS-SSIM Metrics

	MMD (95% CI)	FID (95% CI)	MS-SSIM (95% CI)
medical-3D-DDPM (Ours)	0.036 [0.028–0.045]	19.39 [16.8–22.5]	0.58 [0.51–0.64]
real images	−	−	0.74 [0.68–0.79]

**Fig. 3:**
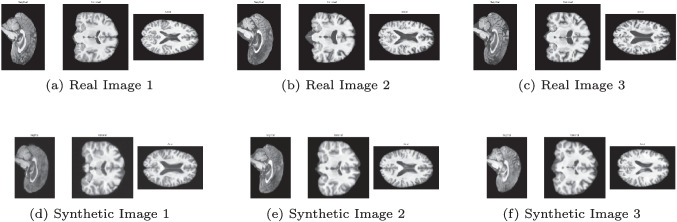
Comparison of real and synthetic MRI shows the diffusion model’s anatomical accuracy

The MMD shows promising preliminary results. The value is very close to 0, indicating that the two distributions are pretty similar. However, the FID is higher, suggesting that the features extracted from the real and synthetic datasets are somewhat different. However, the result is promising, given that this is a preliminary study, as depicted in Fig. 3.

Lastly, MS-SSIM computed on the synthetic dataset is lower than that of the real dataset, indicating that our model generates sufficiently similar brains. In contrast, the structural similarity in the real dataset is higher, suggesting that the brains within it are approximately 16% more similar to each other than those generated by our model.

Expert neuroradiologists from CMND qualitatively reviewed a representative subset of the generated MRIs. Their evaluation confirmed that the synthetic images preserved the overall brain morphology, maintained clear delineation between gray and white matter, and displayed tissue contrasts consistent with those typically observed in real T1-weighted scans. Importantly, no gross artifacts, distortions, or anatomically implausible features were reported, and the spatial proportions of the principal brain structures were judged to be realistic. This independent clinical perspective provides an additional layer of validation, complementing the distribution and perception-based metrics, and underscores that the generated images are not only statistically aligned with real data but also visually consistent with radiological expectations.

## Discussion

This study demonstrates the potential of diffusion models to generate high-fidelity 3D T1-weighted brain MRIs in settings where real data are scarce, such as in rare neurological conditions. Our findings indicate that diffusion-based synthesis can approximate the distribution of real MRI data, offering a viable strategy to mitigate the limitations of small training cohorts in neuroimaging research.

Despite these promising results, important limitations remain. The present work is restricted to T1-weighted MRIs, limiting applicability to multimodal diagnostic workflows that often rely on complementary sequences such as FLAIR or T2. Moreover, the computational demands of volumetric diffusion training require specialized hardware, which is rarely accessible in routine clinical environments. These considerations highlight the need for lighter-weight or latent diffusion approaches that can broaden accessibility and support clinical deployment. In addition, the computational burden associated with training large-scale diffusion models is non-negligible. Future research will explore optimization and energy-aware training strategies to improve efficiency and reduce the environmental footprint of such models. Future research should also emphasise reproducibility through open-model documentation and energy-aware benchmarking to align with transparent and sustainable AI practices. Furthermore, as the present model was trained exclusively on healthy subjects, its generalizability to pathological cohorts remains to be established. Extending the framework to disease-specific data will be essential to assess clinical robustness and practical utility.

From a design perspective, we adopted a scaled linear beta noise schedule to ensure numerical stability during training. Alternative schedules, such as the cosine formulation, may provide benefits in some domains but typically require further tuning or loss modifications, which were beyond the scope of this work. Similarly, the network’s base channel size of 128 was chosen as a pragmatic balance between computational feasibility and generative fidelity: smaller configurations (64 channels) compromised image quality, whereas larger ones (256 channels) yielded only marginal improvements at a steep computational cost. These choices underscore the interdependence of architectural design, resource constraints, and synthesis quality.

Beyond single-value metrics, we reported 95% confidence intervals for MMD and FID, as well as the dispersion of MS-SSIM across sample pairs. This quantification of uncertainty complements mean values and provides a more reliable picture of fidelity and diversity, both of which are essential for data augmentation scenarios where preventing overfitting is as critical as ensuring plausibility. The relatively wide confidence interval observed for FID ([16.8–22.5]) reflects the inherent variability of feature-level similarity measures when applied to limited validation sets of volumetric MRIs. This dispersion highlights the sensitivity of distribution-based metrics to sample size and feature-space representations, underscoring the need for larger evaluation cohorts and complementary perceptual or task-based assessments in future work.

In addition to quantitative evaluations, expert neuroradiologists reviewed a subset of generated MRIs, confirming their overall anatomical plausibility and tissue contrast consistency. While encouraging, this form of expert review should be considered preliminary rather than a systematic clinical validation. Future work must adopt structured radiologist-in-the-loop protocols with standardized rating schemes and inter-rater agreement to ensure reproducibility. Ultimately, the real test of clinical utility will be whether synthetic data demonstrably improves performance in downstream tasks such as segmentation, disease classification, or progression modeling.

Finally, beyond technical and clinical aspects, the responsible integration of synthetic neuroimaging requires attention to ethical and governance issues. Transparent documentation of data provenance, routine bias checks, and clear labeling of synthetic samples will be necessary safeguards. Embedding human expertise into evaluation pipelines and adopting governance tools such as dataset cards will help align generative neuroimaging with regulatory and clinical expectations.

Taken together, these findings suggest that while diffusion models can generate anatomically plausible MRIs with strong quantitative fidelity, significant work remains to achieve systematic clinical validation and ethically responsible deployment. Bridging these gaps will be key to realizing the transformative potential of generative modeling in neuroimaging research and healthcare.

## Conclusion and Future Work

This study introduces a diffusion-based generative framework for synthesizing realistic 3D brain MRI scans, targeting the challenges posed by limited data availability in rare neurodegenerative conditions. Our approach provides a preliminary step toward scalable and clinically meaningful data augmentation in medical imaging.

Building on these results, we will explore latent diffusion strategies to improve output resolution and reduce training costs, as well as incorporate multimodal conditioning to support more complex diagnostic scenarios. We also plan to evaluate the downstream utility of synthetic images in tasks such as classification and segmentation, and involve radiologists in formal human-in-the-loop validation studies to assess clinical plausibility.

Further, we will investigate the latent space of the diffusion model to extract interpretable generative factors associated with neurological conditions. In parallel, we aim to incorporate data-efficient learning strategies, including transfer learning and few-shot learning, to improve model adaptability in low-resource settings typical of rare disease applications.

Finally, we intend to integrate our pipeline into a robust, secure, and regulation-compliant MLOps infrastructure, ensuring traceability, version control, and deployment readiness in real-world clinical environments.

Together, these directions move beyond proof-of-concept, positioning diffusion models as a cornerstone technology for advancing rare disease research and data-driven medicine.

Overall, the results provide preliminary evidence that diffusion models could support scalable and clinically meaningful data augmentation, but further validation on diverse and pathological datasets will be required before safe and responsible translation into clinical workflows.

## Footnotes

1 MONAI: Medical Open Network for AI, https://monai.io/

2 https://github.com/Project-MONAI/MONAI/blob/dev/monai/networks/schedulers/ddpm.py

3 CINECA HPC, https://www.cineca.it/en/
